# Molecullar and biochemical effect of alcohlic extract of Alpinia galanga on rat spermatogenesis process

**Published:** 2014-11

**Authors:** Mahta Mazaheri, Vahid Shahdadi, Ashraf Nazari Boron

**Affiliations:** 1 *Department of Medical Genetic, Faculty of Medicine, Shahid SadoughiUniversityofMedical Sciences, Yazd, Iran.*; 2 *Department of Biology, University of Peyam-Noor, Isfahan, Iran.*; 3 *Institute of Plant Biotechnology, University of Zabol, Zabol, Iran.*; 4 *Department of Physiology, School of Medicine, Zabol University of Medical Sciences, Zabol, Iran.*

**Keywords:** *A. galanga*, *Sperm*, *Spermatogenesis*, *Rat*, *Testis*, *Testosterone*

## Abstract

**Background:**

*Alpinia galanga* (A. galanga) belongs to the Zingiberaceae family has anti-oxidant effects in animals and humans body and often is used as medicament or part of medicaments in Asian folk medicine for various applications.

**Objective:**

The objective of this study was to investigate the molecular and biochemical influence of alcoholic extract from the rhizomes of *A. galangal* on the spermatogenesis process in male rat.

**Materials and Methods:**

Forty five Wistar male rats were divided into three groups, control (n=15) and two tested groups (n=30). Alcoholic extract (5%) of plant was given by oral route at doses of 100 and 300 mg/day for 56 days and spermatogenesis parameters, hormone changes and expression level of the cAMP-responsive element modulator (CREM) gene were assessed.

**Results:**

Methanol extract of A. galanga increased serum testosterones level significantly in both treated groups in comparison with control group (p<0.05). Besides, the percentage of sperm viability and motility in both tested groups were significantly increased. Follicle stimulating hormone FSH hormone, morphology and weight were affected in both treated groups. With 300 mg/day an increase in sperm count was observed. Sperm motility was increased in two treated groups whereas testis weight was decreased in treated groups. Real time analysis of treated cells of testis showed increase level of mRNA related to CREM gene involved in spermatogenesis process after 56 days induction.

**Conclusion:**

It is concluded that application of ethanolic extract of *A.* *galanga* significantly increased sperm percentage, viability, motility and testosterone hormone. This suggested that this plant may be promising in enhancing sperm healthy parameters.

## Introduction

About 13-15% of couples suffer from infertility in the world wherein approximately 40% of the causes are attributed to the woman (1). Male factor is involved in 40-50% of conjugal infertility (2). Several conditions could involve in spermatogenesis process and reduce sperm quality. some factors such as drug treatment, toxins, air pollutions, chemotherapy and inadequate intake of vitamins have harmful effects on spermatogenesis and sperm normal production (3). Several studies have reported that dietary antioxidants can protect sperm DNA from free radicals (4-5). 

The plant species* Alpinia galanga* belongs to the family of *Zingiberaceae *is a perennial herb with rhizomatous root stocks and is mainly known in Asia (China, India, Indonesia, Japan, Malaysia and Thailand), as a drug prepared from the rhizomes and roots and is used as tea or tincture with spasmolytic, antiphlogistic and antibacterial effects, also is used as stomachicum and against gastric diseases (5). 

This species, like other spices is rich in phenolic compounds such as flavonoids and phenolic acids and also rich in alcoholic extract such as cineole, methyl cinnamate, myrecene and contains various flavones such as galangin, alpinin, kampferide and 3-dioxy-4-methoxy (6). The phyto-pharmacological activity of* A. Galanga* as antioxidant and anticancer property has well been reviewed (7-8). Antioxidants protect DNA and important molecules from oxidative damage and can improve sperm quality and consequently enhance fertility rate in men (9). Therefore, the role of nutritional and biochemical factors in reproduction and sub- fertility treatment is very important. The cAMP-responsive element modulator (CREM) is involved in regulating gene expression in spermatogenesis process (10). The CREM gene consists of 14 exons which regulates the production of bot activator and repressor proteins (11). At this time pharmacological investigation has not been performed for the effect of alcoholic extract of species* A. galanga *on spermatogenesis and CREM gene expression. 

In this basic experimental study, we attempted to assess the ability of *A. galanga* to promote sperm parameters and modulate follicle stimulating hormone (FSH), testosterone concentration and CREM gene expression analysis on rat in experimental condition.

## Materials and methods

**Plant material and preparation of alcoholic extract**

This basic experimental study was performed in faculty of medicine of Zabol. The study was approved by the Ethics Committee of the Zabol University of Medical Science, Zabol, Iran. 

The dried rhizomes of *A. galanga *were procured from the local market of Zabol (City in Sistan and Baluchistan in Iran) and were identified in department of biology of Zabol University. The dried galangal powder (1kg dry matter) was extracted with 3L of 80% ethanol and left at room temperature for 24h. The extract was filtered through a Watman filter N.4 paper. The filtrate was collected and concentrated using the rotary evaporator at 55^o^C for 15 min. The dry extract 2.4 gr and 4.8 gr, were dissolved separately in 100 mL of 5% ethanol with resultant solutions of 100 mg/mL (solution A) and 300 mg/mL (solution B). These solutions were kept at 4^o^C until use.

**Animals**

Eight-week-old male Wistar rats purchased from Razi Institute (Mashhad, Iran) and were housed in a specific pathogen-free environment on a 12 hr light/dark cycle at the center for Laboratory Animal Care at University of Medical Sciences of Zabol. Rats were fed Purina laboratory chow (Agribrands Purina Peru S.A, Lima, Peru) (12).

**Treatment with** ***A.*** ***galanga***

Two-months-old male Wistar rats, weighing 175.4±5.8 g, were used for experiment. The rats were divided randomly into three groups of 15 animals each. These three groups, control (treated with normal saline), experimental group1 (treated with solution A; 100 mg/day) experimental group 2 (treated with rhizome extract, 300 mg/day).

**Tissue preparation**

At the end of the treatment period (60 days), the rats were anesthetized with chloroform. The testis was removed, cleared of the adhering tissues and were weighed. The relative testis weight was calculated as the absolute testes weight/body. The epididymis was removed and used for the sperm analysis.

**Sperm count**

For sperm analysis, male rats were divided at random into 3 groups of 10 animals each. Group 1 (Controls) injected 2 mL/day of 5% ethanol. Group 2 (low Alpina treated) received solution A (100 mg/day). Group 3 (high Alpina treated) received solution B (300 mg/day). 56 days after induction, the rats were killed. The testis and epididymides were immediately removed and cleared off the attached fat and connective tissue then were incubated at 37^o^C for 1h (12). Sperm cells with epididymis fluid were diluted with Hanks balanced salt solution and sperm motility and morphology were studied (13). For sperm count, spermatozoa were counted as described by Padmalatha and Vijayalaxmi (14). Sperm suspension was placed on both sides of Neubauer’s hemocytometer and allowed to settle in a humid chamber for 1 hour. The number of sperm in the appropriate squares of the hemocytometer was counted under the microscope of 100 x magnifications.

**Hormone assay**

Blood samples was collected from heart, separated by centrifugation at 3000 rpm for 5 min and blood serum was harvested and stored at -80^o^C to carry out the hormonal assays. Hormone levels were measured by radioimmunoassay coat-A-count kit (diagnostic products corporation, LA, Calif.) using Packard Cobra gamma-counter. Testes were removed, deterged of adhering tissue and weighed.

**RNA isolation and first strand synthesis**

Total cellular RNA was isolated from fresh and frozen tissues by RNeasy Mini Kit (Qiagen, USA.). First-strand cDNA synthesis was carried out in 30 µl of reaction mixture containing 1 µg of total RNA, 6 µl of random hexamers (50 M), 6 µl of 100 mM dithiothreitol (DTT), and 600 U of Moloney murine leukemia virus (MMLV) reverse transcriptase (Qiagene). The reverse transcriptase-negative control was performed without the addition of reverse transcriptase.

**Real-time polymerase chain reaction (qPCR)**

Expression analysis of *CREM* gene involved in spermatogenesis process was performed using real-time PCR (RG3000, Corbett Research). Sequence specific primer for *CREM* (R*: *5-GATTGAAGAAGAAAAATCA GA-3) and (F: 5-TTGACATATTCTTTCTTCTT -3) and GAPDH as reference gene (F: 5'-GCA GCTCCTTCGTTGCCGGT-3'; and R: 5'-CCC GCCCATGGTGTCCGTTC-3') were designed and used to gene expression analysis. 

For real-time PCR, 2 µl of the reverse-transcribed cDNA template (50 ng/µl) was added to a final volume of 20 µl of reaction buffer containing 1.5 mM MgCl_2_, 50 mM KCl, 0.2 mM deoxynucleoside triphosphates, 15 pM each primer, and 0.5 U of *Taq *polymerase in 3 µl of Master *Taq *polymerase enhancer (Eppendorf). 

The real time PCR was done on the three biological and technical replicates. Reaction condition for thermal cycling were: 56 for 2 min, 95 for 5 min followed 40 cycles, 95 for 15 sec and 65 for 1min. PCR products showed a fragment of expected size revealed on agarose gel electrophoresis. Data analysis was performed by REST 2009 software (15).

**Statistical analysis**

The results are expressed as the mean±SD. Analysis of statistical difference between control and samples means was conducted using analyses of variance (one way). P<0.05 was considered significant.

## Results

The results recorded in table I, show that there is a significant change in tested parameter between and within groups. Treated rats were divided to two separate groups according to their weight. Seven morphological and biochemical characters were compared in induced rat with alcohlic extract of *Alpinia galanga* in two separate group I (100 mg/kg/day) and group II (300 mg/kg/day). Our data showed that there is a no significant increasing in testis weight between and within tested groups compared to control group (Table I). Group II revealed more change on measured characters than group I (Table II). 

Results about sperm characteristics showed that treatment of rat with alcoholic extract of *A. galanga* increase sperm count and motility in two experimental groups compared with control group (Table II). Total serum testosterones levels and FSH significantly were increased in both treated groups (p=13) in comparison with control group but progesterone hormone was not significantly increased in treated groups. The control group showed that 91% of spermatozoa had normal morphology. In the control and treated rats in group I (100 mg/day), sperm exhibited slow and non-progressive motility. In group II (300 mg/day) sperm count was significantly increased compared to group I (Table II). 

**Figure 1:**
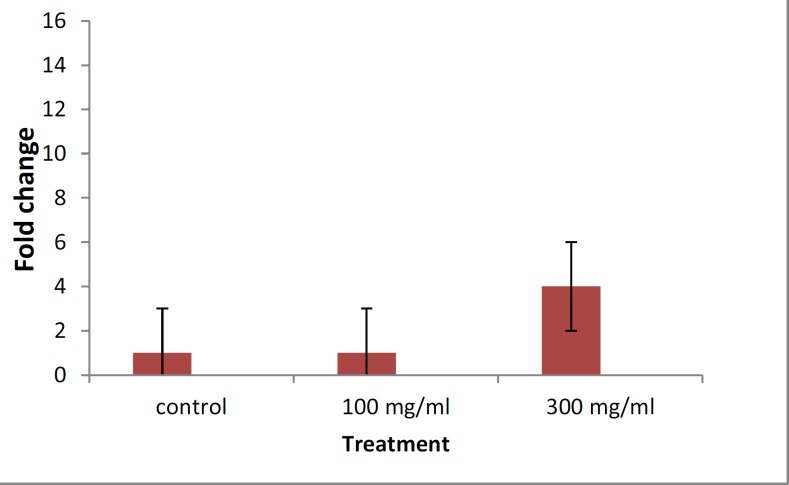
Effect of alcoholic extract of *Alpinia galanga* species on expression level of *CREM* gene involved in spermatogenesis.

Sperm motility in group II after 56 days adding showed rapid and progressive motility. Real time analysis of treated cell of testis by alcoholic extract showed increase level of mRNA related to CREM gene involved in spermatogenesis process after 56 days induction. CREM mRNA levels hadnot significantly difference in testes from the group I (100mg/day) compared to control group. In group II the level of mRNA was incrassated for four folds (Figure 1). These data confirmed the results of morphological and biochemical assays. These results indicate that alcoholic extract of *A. galangal *could have influence on spermatogenesis process.

**Table I:** Analysis of variance of the effect of *A. galangal* on spermatogenesis parameters

**Variance source**	**df**	**Mean Square**							
		**Body weight** **(g)**	**Testis weight** **(g)**	**Sperm count** **(10** ^6^ **/** **ml)**	**Sperm motility** **(%)**	**Testosterone** **(%)**	**FSH hormone** **(%)**	**LH hormone** **(%)**	**Progesterone** **(%)**
Between	2	3541.067*	0.070^ns^	181.529**	955.344 **	17.947 *	1.846 **	0.094^ns^	0.437^ns^
Within	2	807.133	0.061^ ns^	18.521	4.001	4.265	0.079	0.124	3.065
	F-value	4.387	1.145	9.801	238.793	4.207	23.256	0.756	0.143

Ns , * and ** are non-significant and significant at 1% and 5% respectively

**Table II:** Mean comparison of the effect of *A. galanga* on spermatogenesis process at 100 mg/kg and 300 mg/kg

**Variance**	**Average**							
	**Body weight** **(g)**	**Testis weight** **(g)**	**Sperm count** **(10** ^6^ **/** **ml)**	**Sperm motility** **(%)**	**Testosterone** **(%)**	**FSH hormone** **(%)**	**LH hormone** **(%)**	**Progesterone** **(%)**
Control	209.400^b^	1.62	12.950^b^	45.314^c^	2.705^b^	1.223^ b^	1.426	1.962
100 mg/kg	237.400^ab^	1.88	18.800^b^	64.066^b^	6.274^ a^	2.160^ a^	1.167	2.488
300 mg/mg	262.600^ab^	1.842	25.000^a^	72.282^a^	5.593^ a^	2.362^a^	1.375	2.485
LSD	39.15	5.93	5.93	2.756	2.846	0.387	--	--

LSD: Least significance different

## Discussion

This study demonstrated that *A. galanga*l rhizome treatment for 56 days affected sperm parameters and spermatogenesis process in rats. In the present study treatment of rats with the alcoholic extract of *A. galanga*l rhizome causes a significant increase in sperm count and motility. This extract did non causes an increase in testes weight of rats. The effect of this extract on testis weigh is not similar to extract of *Salvia hypoleuca* which increased testis weight of induced rats  (16). This could be due to induction time and modality of extract. 

Toxicity studies on the ethanolic extracts of the rhizomes of *A. galanga* and Curcuma longa in mice shown that these extract has an acute dose from 0.5-3 g/kg body weight while the chronic dosage was 100 mg/kg/day as the extract whiles our data showed alcoholic extract in 300 mg/kg body weight have not chronic effect on studied population (17). The effect of *Matricaria recutita *extract on sperm count and motility of sperm has been studied and showed that this extract has marked reduction in the sperm count and motility compared with control group (16). Analysis of obtained data in our study showed that external morphological and spermatogenic changes in addition to body weight and vital organ weights were increased. These observations are in agreement whit study of Qureish *et al* (17).

These concordance findings suggest that this plant could influence spermatogenesis process in male. The increase in sperm density and motility in cauda epididymis is importance with regard to fertilization (18). Motility of sperm is occurred during their epididymal transit after leaving testis so motility of produced sperm cells from original resource is important in fertility process (19). The effect of the alcoholic extract of *Ruta chalepensis *on the sex organs and hormones of male rats showed an increasing in the cauda epididymis sperm count in the treated animals (20). 

The extract had a direct effect on the testes resulting in an increase in the number of spermatozoa and the increased level of testosterone production (20). Our data also revealed that sperm motility had no significant change in group I but it shows a noticeable increase at group II with high concentration of alcoholic extract of *A. galanga*. Spermatotoxic effects of plants could be observed at lack of motility, decrease sperm count and increase incident of sperm abnormalities condition (20). So we could conclude that extract of studied plant has not toxic effect on spermatogenesis process. This result is similar to results of Gureish *et al* that showed *A. galanga* extract has no spermatotoxic effects (17). 

*CREM *gene expression* and *its related protein have been significantly increased in rat induced by methanolic extract of *Salvia hypoleuca* plant (16). These data are in concordance with our observation. Gene expression analysis showed that this extract is capable to induce CREM gene related to sperm production. The study of antioxidant and anti-diabetic activity of *A. galangal* showed that this extract could increase total protein level and serum triglyceride in treated group when compared to diabetic control (21). These results indicate that this extract could influence protein production via expression of related genes involved in spermatogenesis. 

Results showed that *A. galanga* may enhance male fertility by elevating sperm quality and gene expression so we concluded that *A. galanga*l rhizome extract has spermatogenic activity in adult male rat due to chemical compounds in it and may be useful to produce drugs to improve male fertility. The effect of plant oil or aquatic extract is completely dependent to biochemical characters of extract so comparing the plant extract together need to fractional analysis of extract to detect more effective fragment to use in pharmacological industry.
